# Effects of dexmedetomidine on intraoperative hemodynamics, recovery profile and postoperative pain in patients undergoing laparoscopic cholecystectomy: a randomized controlled trial

**DOI:** 10.1186/s12871-021-01283-z

**Published:** 2021-03-01

**Authors:** Qin Ye, Fangjun Wang, Hongchun Xu, Le Wu, Xiaopei Gao

**Affiliations:** 1 North Sichuan Medical College, No. 234, Fujiang Road, Shunqing District, Nanchong City, Sichuan Province China; 2 Affiliated Hospital of North Sichuan Medical College, No. 63, Wenhua Road, Shunqing District, Nanchong City, Sichuan Province China

**Keywords:** Dexmedetomidine, Laparoscopic cholecystectomy, Cough, Haemodynamic stress response, Postoperative pain

## Abstract

**Background:**

To investigate the optimal dose of dexmedetomidine to maintain hemodynamic stability, prevent of cough and minimize postoperative pain for patients undergoing laparoscopic cholecystectomy.

**Methods:**

One hundred twenty patients were randomly divided into D_1_, D_2_, D_3_ and NS groups, and dexmedetomidine 0.4, 0.6, 0.8μg/kg and normal saline were administrated respectively. Patients’ heart rate, systolic blood pressure and diastolic blood pressure were measured at T_1_-T_7_. The incidence of cough was recorded. Other parameters were noted, the time of spontaneous respiratory recovery and extubation, visual analogue scale scores and dosage of tramadol.

**Results:**

The heart rate, systolic blood pressure and diastolic blood pressure of D_2_ and D_3_ groups has smaller fluctuations at T2–3 and T7 compared with NS and D_1_ groups (*P* < 0.05). The incidence of cough was lower in D_2_ and D_3_ groups than NS group (*P < 0.05*). The visual analogue scale scores and tramadol dosage of D_2_ and D_3_ groups were lower than NS group (*P < 0.05*). The time of spontaneous respiratory recovery and extubation in D_3_ group was longer than that in D_1_ and D_2_ groups (*P < 0.05*).

**Conclusions:**

Intravenous infusion of 0.6μg/kg dexmedetomidine before induction can maintain hemodynamic stability, decrease cough during emergence, relieve postoperative pain of patients undergoing laparoscopic cholecystectomy.

**Trial registration:**

ChiCTR1900024801, registered at the Chinese Clinical Trial Registry, principal investigator: Qin Ye, date of registration: July 28, 2019.

## Background

Patients with general anesthesia are often accompanied with adverse reactions, such as cough, agitation, hypertension and tachycardia, and the incidence of cough is up to 82.5% [1]. The cough during extubation not only brings discomfort to patients, but also leads to hypertension, tachycardia, myocardial ischemia, laryngospasm and other complications. Varieties of methods and drugs have been used in the past to prevent or reduce emergence cough of general anesthesia [1–3]. Studies have found that administration of dexmedetomidine during surgery or at the end of surgery can attenuate stress and cough response, reduce postoperative pain and postoperative nausea and vomiting (PONV). However, with a high dose or administrated at the end of surgery, dexmedetomidine delays awakening and caused bradycardia and other complications [4–8]. For short surgery or day-surgery like laparoscopic cholecystectomy (LC), whether the rational loading dose of dexmedetomidine before induction can attenuate stress and cough response, alleviate postoperative pain and reduce PONV, meanwhile minimize the influence on recovery time and heart rates (HR). Therefore, this clinical trial was designed to investigate the effect of different doses of dexmedetomidine on the quality of anesthesia in patients undergoing LC.

## Methods

### Study design

**Fig. 1:**
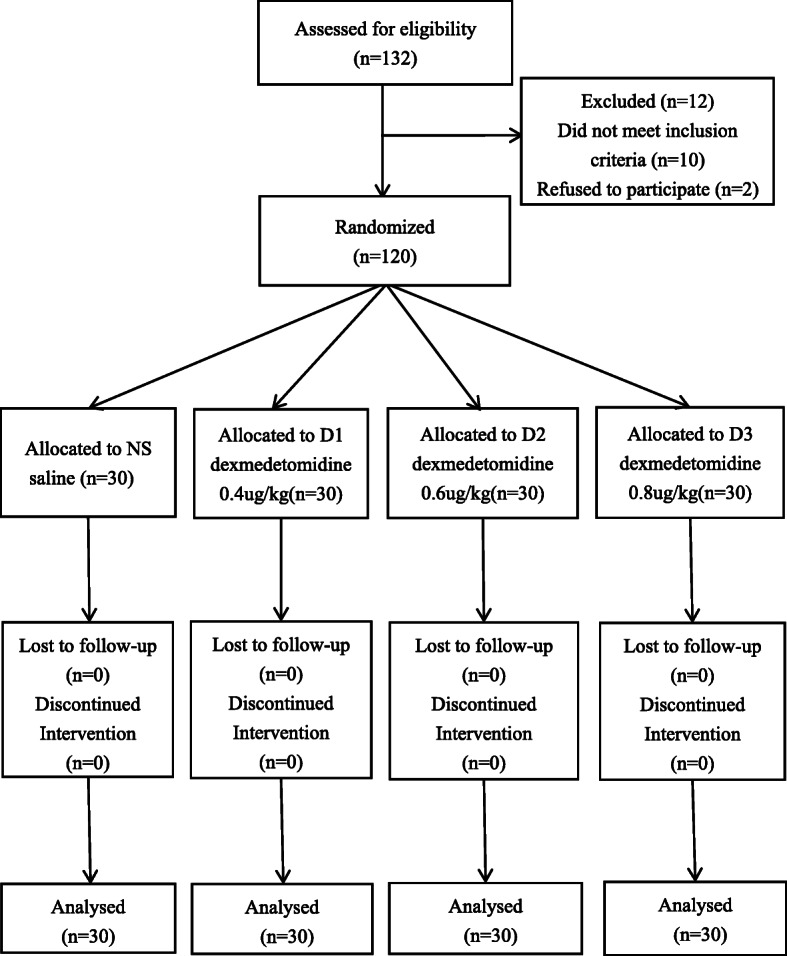
Flow diagram of the study

This study was approved by the Ethics Committee of the Affiliated Hospital of North Sichuan Medical College (2019ER(R)071–01) and registered at the Chinese Clinical Trial Registry (ChiCTR1900024801, Principal investigator: Qin Ye, date of registration: July 28, 2019). All the participants for this prospective, randomized, double-blind, single center clinical trial conducted signed the written informed consents and performed at the Affiliated Hospital of North Sichuan Medical College. All procedures adhered to the applicable CONSORT guidelines (Fig. 1).

All patients were randomized to one of four groups using computer-generated random numbers and a 1:1:1:1 allocation ratio. Marked these random numbers on the cards. Put the marked cards in sealed envelopes in an opaque box. When the patient arrived in the operating room, the anaesthesia nurse randomly drew an envelope and administrated the test drug according to the group in the envelope, which used sealed envelopes indicating the allocation: the same volume of normal saline group (NS group), dexmedetomidine 0.4 μg/kg group (D1 group), dexmedetomidine 0.6 μg/kg group (D2 group) and dexmedetomidine 0.8 μg/kg group (D3 group). The anaesthesia nurse completed the drug preparation and gave it to the anesthesiologist in this study. After the experiment, the anesthesiologist showed the data back to the statistician. Patients, the anesthesiologist and the statistician did not know the grouping, meanwhile anesthesia nurse did not participate in anesthesia management, postoperative follow-up and data analysis.

### Inclusion criteria

One hundred twenty consecutive patients scheduled for elective LC, aged 18–60 years and with 18.5 kg/m^2^ < body mass index (BMI) < 28 kg/m^2^ and ASA physical classification status of I–II, were enrolled from July 2019 to November 2019.

### Exclusion criteria

patients with a history of PONV, motion sickness, gastroparesis, bradycardia, atrioventricular block and severe cardiac dysfunction, diabetes, hypertension, coronary heart disease, liver and kidney function seriously damaged, chronic pain, upper respiratory tract infection, asthma, smoking, allergic to dexmedetomidine. Withdrawal criteria: conversion to open surgery, the operation time over 90 min, massive hemorrhage during surgery, patients refusing to participate.

### Anesthesia

Before surgery, all patients fasted for solid food for 12 h and clear liquids for 4 h, with intramuscular injection of phenobarbital sodium 0.1 g and scopolamine butylbromide 20 mg 30 min in advance. After entering the operating room, the peripheral vein was opened and 10 ml/kg/h lactated ringer solution was administered intravenously. HR, systolic blood pressure (SBP), diastolic blood pressure (DBP), pulse oximetry (SpO_2_), electrocardiography (ECG), end-tidal carbon dioxide (ETCO_2_) and bispectral index (BIS) were monitored. D1, D2 and D3 groups were provided with 10 ml dexmedetomidine containing 4, 6 and 8μg/ml respectively, and the NS group was provided with 10 ml normal saline. Dexmedetomidine or normal saline 0.1 ml/kg was continuously intravenously injected for 10 min and followed by anesthesia induction. The induction of general anesthesia was administrated by intravenous midazolam 0.03 mg/kg, propofol 1.5–2 mg/kg, sufentanil 0.4 μg/kg and rocuronium 0.6 mg/kg. Then tracheal intubation was performed, followed by mechanically controlled ventilation. The pure oxygen flow was 2 L/min, the tidal volume was 8 ml/kg, the respiratory rate was 14 times/min and the inhalation/exhalation ratio was 1:2. Respiratory parameters adjusted according to ETCO_2_ maintained at 35–45 mmHg and SpO_2_ remained above 98%. Intraoperative anesthesia was maintained by sevoflurane and BIS values were remained at 40–60. After induction of anaesthesia for 40 min, 0.2μg/kg sufentanil and 0.2 mg/kg rocuronium were added. Analgesics and muscle relaxant were discontinued 30 min before the end of surgery and inhalation of sevoflurane was discontinued 10 min before. Body temperature of the patients was maintained at about 36 °C during the operation. During surgery, all patients were placed in the position of head upward 30°, left inclination 15°, and abdominal pressure maintained at 12 mmHg. After surgery, the patients met the indications of extubation (call for open eyes and tidal volume > 5 ml/kg), and then the catheter was extracted and transferred to the post-anesthesia care unit (PACU). When the blood pressure decrease was greater than 20% of the base value or SBP decreased to 80 mmHg, ephedrine was given 6-10 mg immediately. When the increase of blood pressure was greater than 20% of the base value or the blood pressure was up to 160/95 mmHg, urapidil 5–10 mg was administrated. When the HR was less than 50 beats per minute, atropine 0.3–0.5 mg was given each time. When the HR was greater than 110 beats per minute, esmolol 10 mg was given. When PONV required medication, ondansetron 4 mg was administrated per time. And when the VAS ≥4, tramadol 2 mg/kg was given.

HR, SBP, DBP were measured and recorded at the time of the patients arriving at the operating room (T1), 1 min before intubation (T2), being intubated (T3), 5 min after intubation (T4), establishing pneumoperitoneum (T5), 5 min after establishing pneumoperitoneum (T6), being extubated (T7) and 5 min (T8) and 20 min (T9) after extubation. To record the incidence of hypotension and bradycardia during the operation, operation time (from cutting skin to dressing), anesthesia time (from anesthesia induction to removing the tracheal tube), spontaneous respiratory recovery time (from stopping inhalation of sevoflurane to spontaneous respiratory recovery) and extubation time (from stopping inhalation of sevoflurane to removing tracheal tube). To assess and record the occurrence and severity of cough during recovery period (grade 0: no cough; grade 1: mild, single cough; grade 2: moderate, frequent cough, lasting time < 5 s, no effect on extubation; grade 3: severe, continuous cough, lasting time ≥ 5 s, affecting extubation) [9]. To mark VAS scores (where VAS 0 = no pain, and VAS 10 = worst pain) and PONV (A 4-point scale:1 = absent; 2 = nausea; 3 = retching; and 4 = vomiting) at 20 min(t1), 2 h(t2), 6 h(t3), 12 h(t4), 24 h(t5), 48 h(t6) after operation. Other indicators were recorded, such as postoperative analgesia dosage, agitation, shoulder pain, sleepiness, dizziness and hoarseness.

### Statistical analysis

A Previous study has shown that the incidence of cough is 66.7% during the tracheal extubation period in the CON group. We hypothesized that dexmedetomidine infusion before induction could reduce the incidence of cough during emergence by 50%. In more general terms, we may have *k* groups. Where *pA* and *pB* represent the proportions in two of the *k* groups. We will compute the required sample size for each of the τ comparisons, and total sample size needed is the largest of these. In the formula below, *n* represents the sample size in any one of these τ comparisons. This calculator uses the following formulas to compute sample size:

$$ n=\left( pA\left(1- pA\right)+ pB\left(1- pB\right)\right)\ {\left(\ \frac{z_1-\alpha /(2r)+{z}_1-\beta }{pA- pB}\right)}^2 $$

$$ 1-\beta =\Phi \left(\mathrm{z}-{z}_1-\frac{\alpha }{2\mathrm{r}}\right)+\Phi \left(-z-{z}_1-\frac{\alpha }{2r}\right),z=\frac{pA- pB}{\sqrt{\frac{pA\left(1- pA\right)}{n}+\frac{pB\left(1- pB\right)}{n}}} $$

Twenty-five patients are required in each group (a power of 80% and α of 0.05). To ensure sufficient sample size, 33 patients were needed for each group.

Statistical analysis was performed by using SPSS 23.0 statistical software. Continuous variables with normal distribution were expressed as mean ± standard deviation ($\overline{x}\pm s$), comparison among groups was performed by one-way ANOVA with a post hoc analysis, comparison at different time points was performed by repetitive measurement and analysis of variance with a Bonferroni correction, and categorial variables was determined by Pearson’s *X*^*2*^ test or *Fisher’s* exact test. *P*-value *< 0.05* was considered to statistically significant.

## Results

We recruited 132 patients to our study, but 10 of them did not meet inclusion criteria and 2 of them refused participation. Thus, 120 subjects were enrolled in our study. After randomization, the participants received respectively 0.4, 0.6 and 0.8μg/kg dexmedetomidine or saline before anesthesia induction. All patients completed the study as shown in Fig. 1.

### Demographic data and clinical characteristicsin

There were no significant differences in gender, age, BMI, operation time and anesthesia time among the four groups (*P > 0.05*). Compared with NS group, the time of spontaneous respiratory recovery and extubation in the D1, D2 and D3 groups were prolonged more significantly (*P < 0.05*). As for comparison among D1, D2 and D3 groups, it was in the D3 group that the time was prolonged more significantly (*P < 0.05*), as shown in Table 1.

**Table 1:** Demographic data and clinical characteristics in four groups

	NS group (*n* = 30)	D1 group (*n* = 30)	D2 group (*n* = 30)	D3 group (*n* = 30)	*P value*
Gender, Female/Male	11/19 (36.7%/63.3%)	12/18 (40.0%/60.0%)	12/18 (40.0%/60.0%)	11/19 (36.7%/63.3%)	1.000
Age (years)	42.6 ± 8.2	42.6 ± 5.9	42.5 ± 7.0	42.6 ± 6.3	1.000
BMI (kg/m^2^)	24.7 ± 2.1	24.5 ± 2.5	23.7 ± 2.7	24.3 ± 2.4	0.411
Duration of surgery (min)	46.5 ± 1.4	49.5 ± 10.2	42.6 ± 13.3	47.3 ± 11.4	0.127
Duration of anesthesia (min)	67.1 ± 11.9	69.8 ± 9.9	62.0 ± 12.4	65.7 ± 15.9	0.119
Spontaneous respiratory recovery time (min)	10.2 ± 1.7	11.9 ± 1.6^*#^	12.3 ± 1.8^*#^	13.8 ± 2.9^*^	0.000
Extubation time (min)	11.8 ± 1.9	13.1 ± 1.4^*#^	13.3 ± 1.4^*#^	16.3 ± 2.6^*^	0.000

Data presented as mean ± standard deviation or numbers (proportion)

*BMI* Body mass index

^*^p < 0.05 vs. NS group; ^#^*p* < 0.05 vs. D3 group

### Perioperative hemodynamic changes

**Fig. 2:**
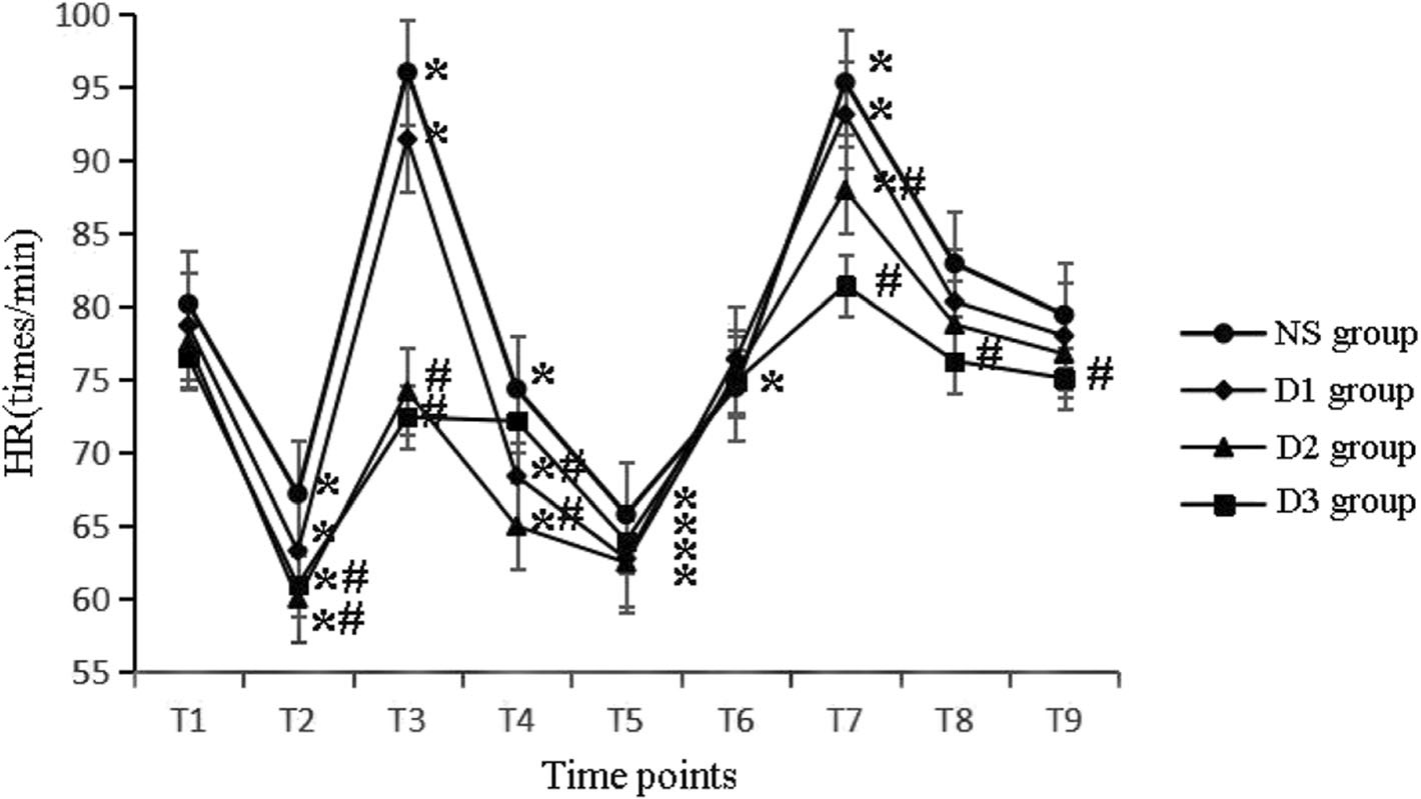
The hemodynamic changes in four groups at different time points

**Fig. 3:**
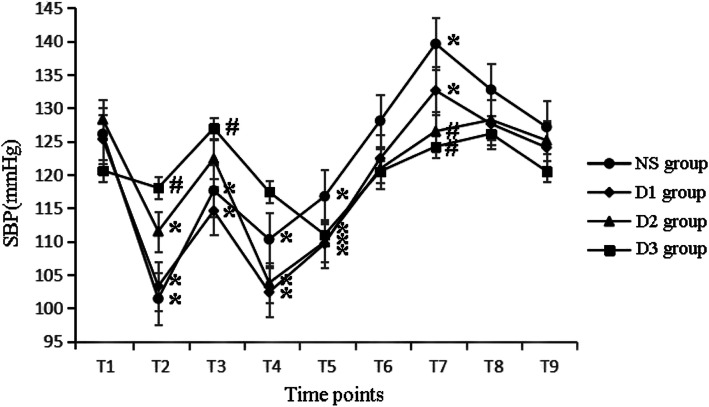
The hemodynamic changes in four groups at different time points

**Fig. 4:**
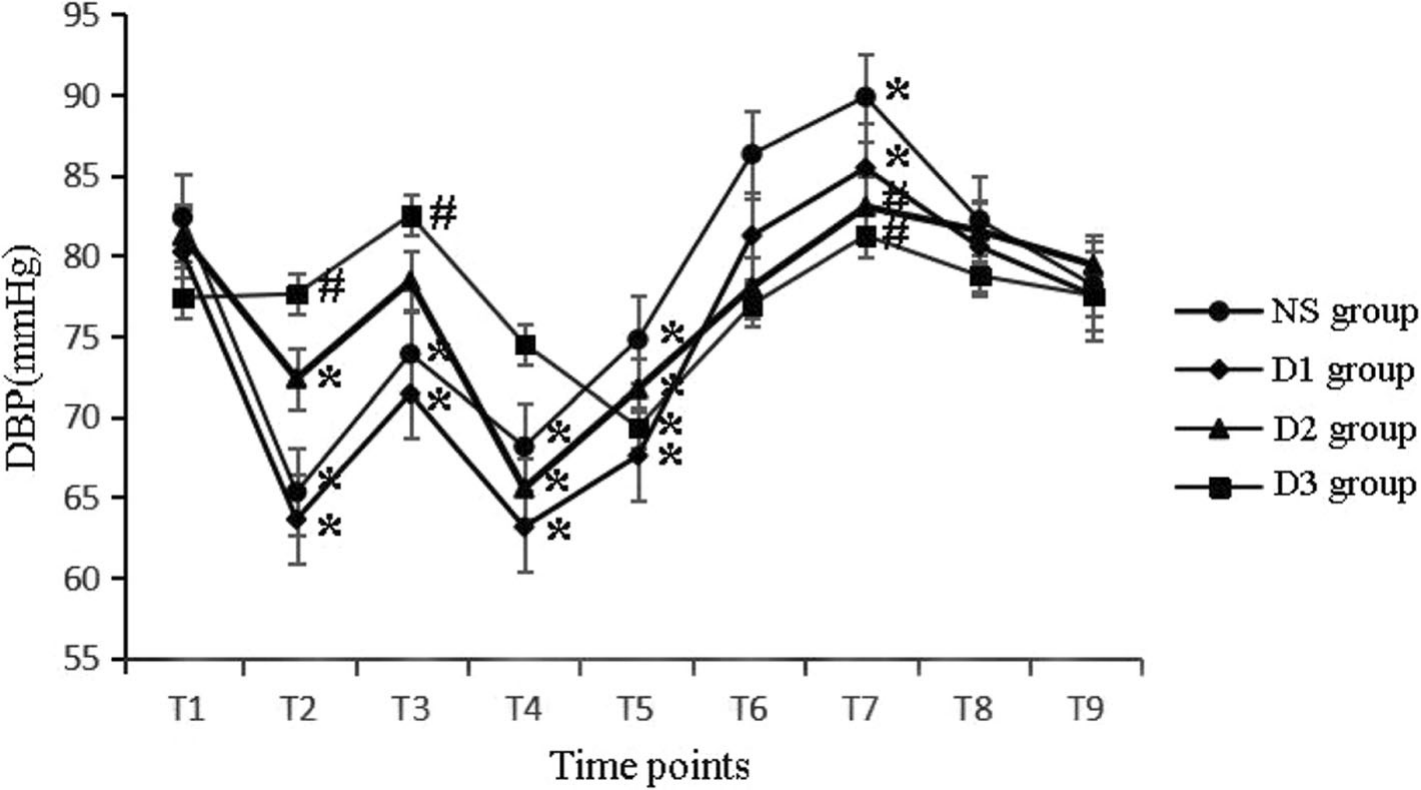
The hemodynamic changes in four groups at different time points

At T1, there were no differences in HR, SBP, DBP among all groups. Compared with T1, HR decreased at T2, T5 in all groups. Besides HR also decreased at T4, T6 in NS group and decreased at T4 in D1 and D2 groups. HR increased at T3 and T7 in NS and D1 groups, while it increased at T7 in D2 group(P<0.05). Compared with NS group, HR decreased at T4 in D1 group, decreased at T2–4, T7 in D2 group and T2–3, T7–9 in D3 group (*P* < 0.05), as shown in Fig. 2. Compared with T1, SBP and DBP decreased at T2–5 and increased at T7 in NS and D1 groups, decreased at T2 and T4–5 in D2 group, decreased at T5 in D3 group (*P* < 0.05). Compared with NS group, SBP and DBP decreased at T7 in D2 group and decreased at T2–3 and T7 in D3 group (P < 0.05), as shown in Figs. 3 and 4.

### The incidence of cough during emergence

Compared with NS group, it was significantly lower in D_2_ and D_3_ groups for the total incidence of cough during emergence (70.00% in NS group vs. 26.67, 23.33% in D2 and D3 groups, respectively, *P<0.008*) and the incidence of moderate cough (56.67% in NS group vs.20.00, 16.67% in D2 and D3 groups, respectively, *P<0.008*). Both the total incidence of cough and the incidence of moderate cough were lower in D1 group than that in NS group, but the differences were not statistically significant (70.00% in NS group vs. 50.00% in D_1_ group; 56.67% in NS group vs. 40.00% in D1 group, *P > 0.05*), as shown in Table 2.

**Table 2:** The incidence of cough in four groups during emergence

	Cough level				Incidence of coughing
	0	1	2	3	
NS group (*n* = 30)	9(30.00%)	4(13.33%)	17(56.67%)	0	21(70.00%)
D1 group (*n* = 30)	15(50.00%)	3(10.00%)	12(40.00%)	0	15(50.00%)
D2 group (*n* = 30)	22(73.33%)*	2(6.67%)	6(20.00%)*	0	8(26.67%)*
D3 group (*n* = 30)	23(76.67%)*	2(6.67%)	5(16.67%)*	0	7(23.33%)*
*P*-value	0.001	0.494	0.002	0	0.001

Data presented as numbers (proportion)

Cough level (grade 0: no cough; grade 1: mild, single cough; grade 2: moderate, frequent coughing, lasting time < 5 s, no effecting on extubation; grade 3: severe, continuous coughing, lasting time ≥ 5 s, affecting extubation)^9^

^*^p < 0.008 vs. NS group

### Comparison of VAS at different time points

At t1–6, the VAS was lower in D2 and D3 groups than that in NS group (*P < 0.05*). At t2–6, it was lower in D2 and D3 groups than that in D1 group (*P < 0.05*). There were no differences between D2 and D3 groups (*P > 0.05*), as shown in Table 3.

**Table 3:** Comparison of VAS at different time points in the four groups (*n = 30,* $\overline{x}\pm s$)

		NS group	D1 group	D2 group	D3 group	*P*-value
VAS	t1	4.0 ± 1.4	3.3 ± 1.5	3.1 ± 1.2^*^	3.2 ± 1.0^*^	0.039
	t2	4.7 ± 1.3	4.7 ± 1.6	3.5 ± 1.5^*#^	3.6 ± 1.2^*#^	0.000
	t3	4.5 ± 1.3	4.2 ± 1.1	3.4 ± 1.5^*#^	3.5 ± 1.1^*#^	0.002
	t4	4.3 ± 1.5	3.8 ± 1.0	3.1 ± 1.6^*#^	3.0 ± 1.1^*#^	0.000
	t5	3.3 ± 1.2	3.2 ± 0.8	2.4 ± 1.3^*#^	2.4 ± 0.9^*#^	0.000
	t6	2.5 ± 0.8	2.3 ± 0.8	1.7 ± 0.8^*#^	1.7 ± 0.7^*#^	0.000

Data presented as mean ± SD

*VAS* Visual Analogue Scale, *t1* 20min after operation, *t2* 2h after operation, *t3* 6h after operation, *t4* 12h after operation, *t5* 24h after operation, *t6* 48h after operation

^*^p < 0.05 vs. NS group; ^#^ *p* < 0.05 vs. D1 group

### The dosage of postoperative analgesic

The dosage of tramadol in D_2_ and D_3_ groups was significantly lower than that in NS and D_1_ groups (152.4 ± 134.6 mg, 127.7 ± 148.1 mg in NS and D1 groups vs.42.5 ± 97.3 mg, 44.3 ± 65.8 mg in D2 and D3 groups, respectively, *P < 0.05*), as shown in Table 4.

**Table 4:** The dosage of postoperative analgesic in four groups (*n* = 30, $\overline{x}\pm s$)

	NS group	D1 group	D2 group	D3 group	*P*-value
The dosage of tramadol (mg)	152.4 ± 134.6	127.7 ± 148.1	42.5 ± 97.3^*#^	44.3 ± 65.8^*#^	0.000

Data presented as mean ± SD

^*^p < 0.05 vs. NS group; ^#^ *p* < 0.05 vs. D1 group

### The incidence of PONV at different time points

The incidence of PONV in NS, D_1_, D_2_ and D_3_ groups were 53.33, 50.00, 46.67 and 40.00% respectively, with no statistically significant differences among the four groups (*P > 0.05*). At t4, the incidence of PONV in D_2_ and D_3_ groups was significantly lower than that in NS group (43.33% in NS group vs. 13.33, 16.67% in D_2_ and D_3_ groups, respectively, *P = 0.033*). There were no differences between D2 and D3 groups (*P > 0.05*), as shown in Table 5.

**Table 5:** The incidence of PONV in four groups at different time points (*n = 30*)

	Different time points					
	t1	t2	t3	t4	t5	t6
NS group	2(6.67%)	12(40.00%)	11(36.67%)	13(43.33%)	6(20.00%)	1(3.33%)
D1 group	1(3.33%)	10(33.33%)	14(46.67%)	8(26.67%)	3(10.00%)	0(0.00%)
D2 group	1(3.33%)	6(20.00%)	11(36.67%)	4^*^(13.33%)	3(10.00%)	2(6.67%)
D3 group	2(6.67%)	6(20.00%)	7(23.33%)	5^*^(16.67%)	2(6.67%)	0(0.00%)
*P value*	0.873	0.218	0.310	0.033^*^	0.406	0.531

Data presented as numbers (proportion)

*PONV* Postoperative nausea and vomiting

^*^p < 0.05 vs. NS group

### The comparison of postoperative adverse reactions

There were no statistically significant differences in the incidence of adverse reactions among the groups (*P > 0.05*), as shown in Table 6.

**Table 6:** The comparison of postoperative adverse reactions among the four groups (*n = 30*)

	NS group	D1 group	D2 group	D3 group	*P*-value
Shoulder pain	8 (26.67%)	6 (20.00%)	5 (16.67%)	3 (10.00%)	0.408
Hypotension	4 (13.33%)	6 (20.00%)	3 (10.00%)	2 (6.69%)	0.446
Bradycardia	0 (0.00%)	3 (10.00%)	4 (13.33%)	5 (16.67%)	0.159
Sleepiness	15 (50.00%)	14 (46.67%)	11 (36.67%)	10 (33.33%)	0.507
Dizziness	9 (30.00%)	11 (36.67%)	12 (40.00%)	8 (26.67%)	0.682
Hoarseness	6 (20.00%)	6 (20.00%)	6 (20.00%)	6 (20.00%)	1.000
Agitation	2(6.67%)	0(0.00%)	0(0.00%)	0(0.00%)	0.107

Data presented as numbers (proportion)

## Discussion

This study found that intravenous infusion of dexmedetomidine 0.6μg/kg and 0.8μg/kg before induction could reduce the stress response during intubation, pneumoperitoneal and extubation in patients undergoing LC, maintain intraoperative hemodynamics more stable, reduce the incidence and severity of cough during extubation, relieve postoperative pain, and decrease both the postoperative analgesic requirements and the incidence of PONV. However, when dexmedetomidine 0.8μg/kg administrated, it delayed the time of spontaneous respiratory recovery and extubation, and significantly increased the incidence of bradycardia. That shows dexmedetomidine 0.6μg/kg may be the optimal dose administered before induction for patients undergoing LC.

Intubation, pneumoperitoneum and extubation during general anesthesia are all harmful stimulus, which can cause a strong stress response. This can lead to increasing the concentration of catecholamines such as epinephrine and norepinephrine in the blood and make the HR and blood pressure elevate [10], which causes a series of complications such as myocardial ischemia, arrhythmia and cerebrovascular accident in patients with cardiocerebrovascular diseases [11]. Intravenous application of dexmedetomidine in the perioperative period can inhibit the release of epinephrine and norepinephrine by activating the receptors in the medullary vasomotor center, thus reduce catecholamine level in the blood by more than 50%, which is beneficial to keep intraoperative hemodynamic stability [12, 13]. Previous study found that continuous infusion of dexmedetomidine 0.2 μg/kg/h or 0.4 μg/ kg/h from 15 min before induction to the end of surgery could reduce the stress response during intubation, pneumoperitoneum and extubation, and the latter was better for maintaining hemodynamic stability with no significant changes in the incidence of bradycardia and hypotension [10]. A single dose of dexmedetomidine 0.5μg/kg or 0.75μg/kg administered before induction of anesthesia can also reduce the stress response during intubation, and there was no significant difference between group 0.5 and group 0.75. However, the incidence of bradycardia and hypotension was significantly higher in 0.75 μg/kg group than that in 0.5μg/kg group [11, 14]. Before the end of the operation, intravenous infusion of dexmedetomidine can alleviate the fluctuation of HR and blood pressure during extubation, and the effect is the best at the dose of 0.5μg/kg with the lowest incidence of bradycardia [5–7]. The results of this study showed that intravenous infusion dexmedetomidine 0.4μg/kg before induction could not effectively inhibit the stress response, but dexmedetomidine 0.6μg/kg and 0.8μg/kg could effectively restrain the intubation reaction, attenuate the intraoperative stress response, and maintain the hemodynamic stability. However, we found that the incidences of bradycardia in the groups dexmedetomidine 0.4μg/kg, 0.6μg/kg and 0.8μg/kg were 10.00, 13.33 and 16.67% respectively, indicating that the incidence of bradycardia increased with the increase of dexmedetomidine dose. Seo KH et al. also found that the incidences of bradycardia at 0.75μg/kg and 1μg/kg increased compared with that at 0.5μg/kg [15], which was consistent with our finding. The occurrence of bradycardia is related to the inhibition of atrioventricular node and sinoatrial node function, reduction of catecholamine content in the blood and excitation of vagus nerve by dexmedetomidine [12, 16].

Cough during the recovery period of general anesthesia is a more concerned problem, mainly caused by the stimulation of endotracheal tube, secretions and volatile anesthetics, which not only brings unpleasant feelings to patients, but also accompanies with complications such as laryngospasm, circulation fluctuation, arrhythmia, wound dehiscence and bleeding. Many drugs such as propofol, ketamine, remifentanil and lidocaine have been used to reduce the cough reflex during extubation [1–4]. Dexmedetomidine is a α_2_ adrenergic receptor agonist that can produce sedative and anti-anxiety effects through receptors in the locus coeruleus without respiratory depression [12, 17]. Moreover, it is often used to reduce cough during the emergence of general anesthesia due to its unique sedative effect [2, 4, 5]. However, the dose-effect relationship is still controversial. Previous studies [6, 7] found that continuous infusion of 0.5μg/kg dexmedetomidine 10 min before suturing skin could reduce the incidence of cough, but the incidence was still up to 64–70%. Intravenous infusion of 1μg/kg dexmedetomidine at the end of operation could reduce the incidence and severity of cough in the recovery period, while 0.5μg/kg dexmedetomidine had no significant inhibitory effect on cough [5]. This showed that the incidence of cough had relation to the dosage of dexmedetomidine. Our study found that the incidence of cough in NS group was 70.00%, while dexmedeidine 0.4μg/kg, 0.6μg/kg and 0.8μg/kg groups were 50.00, 26.67 and 23.33% respectively. It showed that there was a positive correlation between the incidence of cough and the dose of dexmedetomidine. But there were no obvious differences between 0.6μg/kg and 0.8μg/kg dexmedetomidine. In this study, the incidence of cough following intravenous infusion of dexmedetomidine 0.8μg/kg and 0.6μg/kg before anesthesia induction was lower than that in the previous study [5–7]. This inconsistency may be because the time of thyroid surgery was longer than LC and the judgment of cough was different, which was based on the head movement of patients. Our experimental judgment is based on the patients’ cough.

Although the trauma of LC is small, postoperative pain is still the main reason that affects postoperative recovery and prolongs hospital stay. Previous studies have shown that dexmedetomidine could effectively relieve postoperative pain and improve the quality of postoperative recovery [8, 18]. Because dexmedetomidine reduced inflammatory mediators and substance P caused by surgical trauma [8, 12]. A meta-analysis [19] showed that dexmedetomidine could relieve postoperative pain and reduce the dosage of postoperative analgesic, but the optimal dose of dexmedetomidine needs further study. This study found that intravenous infusion of dexmedetomidine 0.6μg/kg and 0.8μg/kg before induction could significantly reduce VAS scores and postoperative analgesic requirements, with no significant differences between the two groups. However, another study [20] showed that a bolus of dexmedetomidine 1 μg/kg preoperatively administered, followed by a continuous infusion of 0.5 μg/kg/h, could significantly reduce the postoperative analgesic consumption, but had little effect on VAS scores. That may be related to the small sample size and local anesthetics wound infiltration before pneumoperitoneum.

Previous studies [21] have showed that intravenous infusion of dexmedetomidine 1μg/kg before operation could reduce the overall incidence of PONV in patients undergoing LC. In this study, we found that dexmedetomidine had no significant effect on the overall incidence of PONV. It was mainly related to dexmedetomidine with a low dose in the study. However, we found that the occurrence of PONV peak in patients with LC was from 6 h to 12 h after surgery, and the incidence of PONV in this period could be significantly reduced by dexmedetomidine 0.6μg/kg or 0.8μg/kg. The incidence of shoulder pain in the dexmedetomidine 0.4μg/kg, 0.6μg/kg and 0.8μg/kg groups (20.00, 16.67 and 10.00%, respectively) were lower compared with NS group (26.67%), indicating that dexmedetomidine could reduce the incidence of postoperative shoulder pain in patients after LC, which also has positive correlation with dose. This may be related to dexmedetomidine’s analgesic and anti-sympathetic effects. In addition, the research also found that the incidence of postoperative sleepiness in the NS group was 50%, while there were respectively 46.6, 36.67, and 33.33% in the dexmedetomidine 0.4μg/kg, 0.6μg/kg and 0.8μg/kg groups, which suggested that intravenous infusion of dexmedetomidine before induction could reduce the incidence of postoperative sleepiness. This is mainly because dexmedetomidine reduces the use of anesthetics and analgesics during operation [19]. Previous studies have found that dexmedetomidine could reduce the incidence of agitation during the recovery period by 37–46% [8, 18]. In this study, the incidences of agitation in the dexmedetomidine groups were compared with the group NS (0% vs. 6.67%), showing that dexmedetomidine can reduce the incidence of postoperative agitation because its effects of sedative, analgesic and anti-anxiety [8, 12, 18].

This study showed that the spontaneous respiratory recovery time and extubation time increased more significantly in the experimental groups compared with NS group. The dexmedetomidine 0.8μg/kg group had the greatest effect on the spontaneous breathing time and extubation time, which was similar to previous studies [4, 6–8, 10, 11, 14, 15, 17]. The higher the dose of dexmedetomidine, the greater the effect on the spontaneous breathing time and extubation time of patients. This may be related to “co-sedation” rather than over sedation of dexmedetomidine [8]. Laparoscopic cholecystectomy is a short operation and the operation time is generally about 30 min. Continuous infusion of dexmedetomidine during surgery further affected postoperative recovery time and extubation time compared with preoperative bolus infusion. In the previous study, it was found that patients with continuous intravenous infusion of dexmedetomidine 0.5μg/kg/h were still under deep sedation 15 min after entering PACU [22]. This is also the reason for using slow bolus infusion (10 min) rather than continuous infusion during surgery in this study.

There are several limitations in our study. First, intraoperative hemodynamic changes informed the grouping, which might influence the assessment of cough. Second, the sample size was calculated according to the incidence of cough during recovery period, so further study was needed to determine if there was statistical significance among other observation indicators. Third, the dosages of anesthetics and analgesics during the operation was not counted in this study, so the effects of different doses of dexmedetomidine on the dosages of anesthetics and analgesics in operation were unclear. Fourth, the dosage of tramadol in D2 and D3 groups was significantly lower than that in NS and D1 groups. That maybe effect the incidence of nausea and vomiting. In future studies on the effect of dexmedetomidine on postoperative nausea and vomiting, this interference factor should be avoided.

## Conclusion

The administration of 0.6μg/kg dexmedetomidine before anesthesia induction can attenuate the stress response during intubation, pneumoperitoneum and extubation, maintain the hemodynamics more stable, reduce the incidence and severity of cough during emergence period, relieve postoperative pain, decrease postoperative adverse reactions such as PONV, shoulder pain, sleepiness and agitation, and have less effect on the spontaneous breathing time and extubation time.

## Data Availability

The datasets used and/or analysed during the current study are available from the corresponding author on reasonable request.

## Abbreviations

NS group: The same volume of normal saline group
D1 group: Dexmedetomidine 0.4 μg/kg group
D2 group: Dexmedetomidine 0.6 μg/kg group
D3 group: Dexmedetomidine 0.8 μg/kg group
PONV: Postoperative pain and postoperative nausea and vomiting
VAS: Visual analogue scale
LC: Laparoscopic cholecystectomy
HR: Heart rates
SBP: Systolic blood pressure
DBP: Diastolic blood pressure
SpO_2_: Pulse oximetry
ECG: Electrocardiography
ETCO_2_: End-tidal carbon dioxide
BIS: Bispectral index
PACU: Post-anesthesia care unit
